# Expression of Editorial Concern, Correction of Conflict of Interest and Affiliation, and Data Corrections

**DOI:** 10.2196/publichealth.9932

**Published:** 2018-05-11

**Authors:** JMIR Editorial Office

## Expression of Editorial Concern

We are hereby expressing serious concerns over conflicts of interests (COI) by the authors of a JMIR Research Protocols paper [1] as well as a subsequent JMIR Public Health & Surveillance [2] paper (where the results of the protocol were published). While we think that complementary medicine (as well as associated lifestyle changes, technologies promoting such lifestyle changes, and other tools and behavioral innovations covered by JMIR journals) deserve to be evaluated in a scientific and evidence-based manner, it is important that these evaluations are performed either by independent researchers who do not have a stake in the outcome, or—if factors exist that could be perceived as conflict of interest—any such potential conflicts of interests are fully disclosed and properly managed from the outset (such as data vetting by an independent party). In the case of these two papers, the initial disclosure was incomplete, and it is unclear to what degree the competing interests of the authors were properly managed when the research was conducted.

In the JMIR Research Protocols [1], and subsequent results paper [2], authors describe a comparative analysis comparing a group of people associated with a specific “complementary medicine health care organization” (Universal Medicine, UM), with the general population, concluding in their results paper that the UM group has “unusual health indicators” (more favorable than the general population).

Both submitted manuscripts originally contained conflict of interest (COI) statements which read as follows:

CS and VM are insiders in that they attend Universal Medicine events. However, they have received no funding, reimbursement, or other consideration from UM or its stakeholders, and no instructions or directions of any kind from UM or its stakeholders. No other competing interests exist.

After acceptance, our freelance copyeditor edited this statement out and replaced it with our standard verbiage “Conflicts of Interest: None Declared,” which is used when there is no COI, because “attending events” in itself is not normally something that would be considered a conflict of interest requiring disclosure. Authors did not object to these copyediting changes and approved the galley proofs. Their signed “license to publish” does not contain any further COI disclosures.

Shortly after publication, we received a 12-page letter from a third party whistleblower, detailing extensive undisclosed conflicts of interests of the authors, which made clear that their COIs go way beyond being “insiders in attending Universal Medicine events.” The letter was also addressed to another journal which published another protocol of the group [3], as well as to the University of Queensland (the lead author CS is associated with that university in his capacity as a PhD student).

We asked authors to provide a more detailed conflict of interest statement for a possible correction of the original papers.

In response, the lead author submitted a 1-page revised COI statement (see below) detailing that all four authors have varying degrees of association with Universal Medicine and are—most significantly—members of the “Practitioners’ Association” which is the body regulating practitioners who are “qualified to practice Universal Medicine modalities.” Of special significance is that two authors have “occasionally offered paid private healing sessions.”

The revised COI by the author also claims that “all authors have experienced substantial health benefits since they started visiting UM events.” In addition, they all have published blogs on UM associated websites. The wife of the lead author is—according to the revised COI—involved in “voluntary activities around producing content” for a UM-associated company and is a “company secretary” of the UM-associated company Unimed Living (owned through another company by the UM-founder Serge Benhayon)  and “does this in an honorary capacity. She is not a director or shareholder” and “does not receive any financial incentives” from UM.

We consulted the original peer-reviewers of the results paper showing them the updated COI and they stated they would not have accepted the manuscript would they have known about these extensive COIs.

We suggested to the authors that we feel that given the significant COIs (as well as the statistical errors in the results paper, which inflated the effect sizes) both articles should be retracted and we would prefer to do this with their consent. The lead author rejected this with the argument that they originally submitted a conflict of interest which the journal removed. We maintain that the original COI submitted stating that two authors “attend UM events” was inadequate and unclear, and did not cover the full extent of the COI. The lead author CS also maintains that the involvement of his wife as company secretary for a Universal Medicine company is irrelevant because it is not a paid position. We checked the company registration documents of Unimed Living and CS’s spouse is indeed listed as company secretary, which is considered an “officer” of a corporation in Australia, so this is not just a merely administrative position, rather, they have many of the same duties and obligations as directors [4]. Thus—even in the absence of remuneration—such involvement constitutes a significant COI.

We remind our authors of the fact that “The potential for conflict of interest can exist whether or not an individual believes that the relationship affects his or her scientific judgment.” [5] and that—while financial relationships are the easiest to identify—conflicts can occur for other reasons, such as religious beliefs, personal relationships, and intellectual passion.

Our concerns with the COI of the lead author (and his spouse) go beyond financial COIs, as in his blog the lead author describes how meeting the UM founder “changed our lives profoundly” [6], and his spouse is describing “seemingly miraculous changes” [7] as a result of UM. This level of “passion” for UM and their involvement may affect the authors’ scientific judgement.

The University of Queensland has launched an investigation, but the investigation is (as of May 11th, 2018) not complete. In the meantime, we are publishing the updated COI statement as corrigendum and this statement of editorial concern, while we await the outcome of the university investigation to decide on further steps.

We are furthermore concerned about the fact that the authors recently also requested the removal of the University of Birmingham as affiliation of one co-author (JK), which is a unusual request.

While authors never claim otherwise, we should stress that the proposed [1] and executed research [2] does not provide any evidence that any Universal Medicine modalities are effective in making people healthier. There are severe limitations regarding on what can be concluded from an observational, cross-sectional study without a control group. One possible explanation for why UM members are apparently healthier than the rest of the population is simply selection bias, meaning that people being associated with UM were always healthier, or less healthy when they joined UM, with "regression to the mean" over time. Another possible explanation involves confounding factors, or the simple fact that UM members adopt healthier lifestyles.

*G. Eysenbach*

*Editorial Director, JMIR Publications*

## Authors’ Corrigendum

(as submitted by the authors)

### Affiliation

The authors request to change Jane Keep's affiliation to:

The Leaders Leader, Greater London, United Kingdom

Instead of:

Health Services Management Centre, School of Social Policy, University of Birmingham, Birmingham, United Kingdom

### Conflicts of Interest

The authors were advised to change the conflict of interest statement. The new Conflict of Interest Statement should read as follows:

All four authors have varying degrees of association with Universal Medicine and are currently members of the Esoteric Practitioners’ Association (EPA) which is the body regulating practitioners who are qualified to practice Universal Medicine modalities.

Universal Medicine has a focus on complementary-to-medicine practices, that aim to support and augment medical treatments.

Jane Keep has attended Universal Medicine workshops since October 2003. Jane Keep was a director of Universal Medicine UK until 2013. She is a member of the EPA, and a committee member of the EPA, and has been accredited by the EPA to offer Esoteric Healing Modalities since 2010. From 2009-2012 Jane ran a small clinic in England which offered Universal Medicine healing modalities.  Since 2012 Jane has been working in corporates/universities/hospitals and occasionally offered paid private Esoteric Healing sessions, though since 2014 she has offered no paid private Esoteric Healing sessions. She was a contributor to Unimed Living 2013 – 2016. Jane has a PhD which referenced the work of over 300 people including Serge Benhayon.

Eunice Minford is a Consultant General Surgeon, and has trained as an Interfaith Minister and Spiritual Counsellor. She also attended the National University of Ireland and obtained a degree of “Master of Applied Christian Spirituality” studying Sacred Esoteric Healing in her thesis. Eunice is also editor of the website “Medicine and Serge Benhayon” and a contributor to that website and to the “Unimed Living” website. She has her own blog “The Soulful Doctor” where she discusses, et al, Universal Medicine. She is also on the EPA professional committee as well as a medical advisor to, and the International Patron of, the EPA. She is a trained esoteric healing practitioner and provides occasional private sessions.

Christoph Schnelle is a financial adviser and has some Universal Medicine associated persons among his client base. Christoph is currently working towards his PhD with The University of Queensland, the subject of which is two randomised controlled trials of Esoteric Connective Tissue Therapy (a Universal Medicine modality) on chronic low back pain and has accumulated case studies as part of this project. Christoph Schnelle’s wife, Nicola Lessing, is involved in voluntary activities around producing content for “Unimed Living” and other websites. Nicola is company secretary of Unimed Living and does this in an honorary capacity. She is not a director or shareholder of Unimed Living. She is not employed by Universal Medicine or Unimed Living and does not receive any financial incentives from Universal Medicine or Unimed Living.

Vanessa McHardy is involved in voluntary activities around producing content for "Unimed Living”, presenting at a conference on Psychological Well Being in 2013 on the Gold Coast of Australia. She has no other involvement other than what is set out below.

All four authors have experienced substantial health benefits since they started visiting Universal Medicine events. They all have published blogs on Universal Medicine associated websites and all four have commented on other blogs published on those websites.

All four have no financial ties and have received no money from Universal Medicine or its related entities including no reimbursements of expenses. Each one attends more than 10 Universal Medicine events a year and regularly receive treatments from Universal Medicine accredited practitioners.

### Data

In some places the standard error was used instead of the correct standard deviation.

The abstract should read:

Differences and corresponding effect size estimates (Cohen *d*; ≥0.8 is a high difference, ≥0.5 a medium and ≥0.2 a small one with *P*<.001 except where indicated) included body mass index (BMI; 1.11), stress level (0.20, *P*=.006), depression (0.44), summary physical (0.31) and mental health (0.37), general mental health (0.39), emotional (0.15, *P*=.009) and social functioning (0.22), vitality (0.58), and general health (0.49), as well as lower incidences of diabetes, hypertension, and thrombosis (*P*<.001 each).

Instead of:

Differences and corresponding effect size estimates (Cohen *d*; ≥0.8 is a high difference) included body mass index (BMI; 10.8), stress level (0.64), depression (4.4), summary physical (4.6) and mental health (5.1), general mental health (7.6), emotional (4.5) and social functioning (4.9), vitality (11.9), and general health (10.1), as well as lower incidences of diabetes, hypertension, and thrombosis (*P*<.001 each).

From the Data Analysis:

We calculated Cohen *d* using the Stata command esizei (version 14.2; StataCorp LLC) using the pooled standard deviation [30] with Welch’s approximation.

Should be changed from:

We calculated Cohen *d* using the Stata command esizei (version 14.2; StataCorp LLC). The ALSWH standard deviations are reported to only 1 significant digit, so we assumed the maximum possible standard deviation (eg, 0.1 became 0.149 and 0.0 became 0.049, reducing Cohen *d*), from which we used the pooled standard deviation [30] with Welch’s approximation.

From Results:

Observed effect sizes (Cohen *d*) ranged from 0.20 to 1.11 (Table 3) [33,34].

Should be changed from:

Observed effect sizes (Cohen *d*) ranged from 0.6 to 11.9 (Table 3). These values are with one exception higher than the 0.8 considered to denote a large effect [33,34].

The new version of Table 3 with amended Cohen *d* and *r* is as below:

**Table 3:** Results from standard survey scales in the Australian Longitudinal Study on Women’s Health (ALSWH) and Universal Medicine (UM) groups, with *r* values and standard deviation.

		Survey respondents with ages covered by ALSWH surveys				ALSWH respondents with UM frequency weights			Effect size			
		n^a^	Mean	95% CI	SD^b^	Mean	95% CI	SD^b^	Cohen	95% CI	*r* ^c^	*pc*
Body mass index (kg/m^2^)		253	21.0	20.7-21.4	2.97	26.1	25.9-26.2	4.6	1.11	0.98-1.23	.48	4*10^-66^
Stress^d^ (lower is better)		200	0.63	0.55-0.70	0.52	0.73	0.72-0.75	0.53	0.20	0.057-0.38	.10	0.0059
Perceived Control Scale^d^		135	4.9	4.8-5.0	0.63	4.3	4.3-4.3	0.79	0.74	0.57-0.91	.35	2*10^-17^
CES-D^d^ (lower is better)		233	3.6	3.1-4.2	5.60	6.1	6.1-6.2	5.6	0.44	0.31-0.57	.21	6*10^-11^
**SF-36^d^**												
	Summary Physical Health	272	52.8	51.9-53.6	10.0	49.7	49.4-49.9	10.0	0.31	0.19-0.43	.15	6*10^-7^
	Summary Mental Health	272	51.4	50.4-52.5	10.0	47.7	47.5-47.9	10.0	0.37	0.25-0.50	.18	10^-9^
	General Mental Health	295	80.1	78.5-81.7	13.6	73.2	72.9-73.4	17.9	0.39	0.27-0.51	.19	5*10^-11^
	Role Emotional	294	85.3	82.2-88.3	26.5	79.6	79.2-79.9	36.9	0.15	0.038-0.27	.08	0.0091
	Social Functioning	295	87.1	84.9-89.3	19.1	81.9	81.7-82.1	24.0	0.22	0.10-0.33	.11	0.0002
	Vitality	295	69.5	67.6-71.5	17.2	57.5	57.2-57.8	20.7	0.58	0.47-0.70	.28	9*10^-23^
	General Health	275	81.9	80.0-83.8	15.9	71.8	71.6-71.9	20.9	0.49	0.36-0.61	.24	3*10^-15^
	Bodily Pain	294	82.8	80.6-85.0	19.5	70.7	70.4-70.9	24.0	0.51	0.39-0.62	.25	2*10^-17^
	Role Physical	294	84.8	81.6-88.0	27.9	78.2	77.9-78.6	36.2	0.18	0.10-0.34	.09	0.0019
	Physical Function	294	89.5	87.9-91.0	13.3	84.6	84.1-85.1	19.7	0.25	0.16-0.40	.12	0.00003

^a^Number of UM respondents with ages that were surveyed in ALSWH for this particular question.

^b^SD: standard deviation.

^c^The r value was calculated as r=d /(sqrt[4+ d^2^]), where *d* is Cohen *d* as derived from the formula given by Nakagawa and Cuthill [32]. *P* value calculated with Satterthwaite’s *t* test.

^d^Multi-item summed scores for perceived stress, Perceived Control Scale, Center for Epidemiologic Studies Depression Scale (CES-D), and 36-Item Short Form Survey (SF-36) using Australian coefficients.
